# Subgroup and sensitivity analyses of annualized relapse rate over 2 years in the ADVANCE trial of peginterferon beta-1a in patients with relapsing-remitting multiple sclerosis

**DOI:** 10.1007/s00415-016-8182-4

**Published:** 2016-06-17

**Authors:** Scott D. Newsome, Bernd C. Kieseier, Douglas L. Arnold, Shulian Shang, Shifang Liu, Serena Hung, Guido Sabatella

**Affiliations:** 1 Department of Neurology, Johns Hopkins University, Baltimore, MD USA; 2 Department of Neurology, Medical Faculty, Heinrich-Heine University, Düsseldorf, Germany; 3 Biogen, Cambridge, MA USA; 4 Montreal Neurological Institute, McGill University, Montreal, QC Canada; 5 NeuroRx Research, Montreal, QC Canada; 6 Pathology 627, Johns Hopkins Neuroimmunology and Neuroinfectious Diseases, Johns Hopkins Hospital, 600 N. Wolfe St., Baltimore, MD 21287 USA

**Keywords:** Interferon, Pegylated, Peginterferon beta-1a, Multiple sclerosis, Relapsing-remitting, Clinical trial, Phase 3

## Abstract

ADVANCE was a 2-year, double-blind, placebo-controlled, Phase 3 study in 1512 patients aged 18–65 years with relapsing-remitting multiple sclerosis, which demonstrated that peginterferon beta-1a 125 mcg administered subcutaneously every 2 or 4 weeks led to significant reductions in annualized relapse rate (ARR) compared with placebo. This analysis examined ARR over 2 years in ADVANCE across subgroups. Patients were treated with peginterferon beta-1a every 2 weeks or every 4 weeks, or placebo during Year 1. Thereafter, patients on placebo were re-randomized to peginterferon beta-1a every 2 weeks or every 4 weeks (delayed treatment). Subgroup analyses were conducted by demographics and baseline disease characteristics. The following results compared ARR in these subgroups for patients in continuous 2-week treatment versus continuous 4-week treatment. ARR was similar in most demographic and baseline disease characteristic subgroups evaluated within the peginterferon beta-1a every-2-week arm or every-4-week arm over 2 years. Although for both doses some differences in the point estimates for ARR were noted among the subgroups, considerable overlap in the confidence intervals suggested that the efficacy of peginterferon beta-1a is similar in all patients irrespective of gender, age, body weight, geographical region, and disease activity at initiation of treatment. Within each peginterferon beta-1a dosing group, ARR was generally similar across most subgroups.

## Introduction

Peginterferon beta-1a is a new treatment for relapsing-remitting multiple sclerosis (RRMS) with the advantage of a more convenient, low frequency, subcutaneous (SC) dosing regimen compared with other beta interferons [1, 2]. The ADVANCE study was a 2-year, multicenter, randomized, double-blind, parallel-group trial, with a 1-year placebo-controlled period, designed to evaluate the safety and efficacy of SC peginterferon beta-1a 125 mcg administered once every 2 or 4 weeks in patients with RRMS (ClinicalTrials.gov number NCT00906399).

Primary results and 2-year follow-up data for the whole study population (*N* = 1512) have been published [3, 4]. At the end of the placebo-controlled period (Year 1), both peginterferon beta-1a dosing regimens met the primary endpoint [reduced annualized relapse rate (ARR)]. Peginterferon beta-1a every 2 weeks resulted in a numerically greater treatment effect across relapse activity outcomes and magnetic resonance imaging (MRI) endpoints than peginterferon beta-1a every 4 weeks [3]. ARR was further reduced in Year 2 with every-2-week dosing, and maintained with every-4-week dosing [4].

The safety profile of peginterferon beta-1a was similar across both dosing regimens and placebo in the first year [3], and adverse events (AEs) were consistent with those of the established disease-modifying treatments for RRMS in the interferon-beta class, with injection-site reactions (ISR) and flu-like symptoms (FLS) being the most commonly reported AEs [5–8]. The safety profile during the non-placebo controlled second year of active treatment was consistent with Year 1 in terms of incidence, severity, and most common AE [3, 4].

To test whether the favorable efficacy reported in ADVANCE is consistent across different types of patients in the RRMS study population, we performed a pre-specified subgroup analysis of ARR at Year 1 which was repeated using data over 2 years. Sensitivity analyses were also conducted to assess the robustness of the primary endpoint results.

## Methods

### Study design and patients

The study design and patient population of the ADVANCE study have been described in detail previously [3]. In brief, patients aged 18–65 years who had been diagnosed with RRMS (McDonald criteria 1–4), had a baseline Expanded Disability Status Scale (EDSS) score of ≤5.0, and had experienced ≥2 relapses within the last 3 years (including ≥1 relapse in the 12 months prior to randomization) were included in the study. Patients were randomized 1:1:1 to receive self-administered SC treatment with placebo, peginterferon beta-1a 125 mcg every 2 weeks, or peginterferon beta-1a 125 mcg every 4 weeks. To maintain blinding, all patients received an injection every 2 weeks; patients in the peginterferon beta-1a every-4-weeks group had alternating injections of peginterferon beta-1a 125 mcg and placebo. At the end of Year 1, patients in the placebo group were re-randomized to SC peginterferon beta-1a every 2 weeks or every 4 weeks (delayed treatment). Patients were excluded if they had progressive forms of multiple sclerosis (MS) or had received prior interferon beta treatment for RRMS exceeding 4 weeks or discontinued interferon treatment within 6 months prior to baseline.

The ADVANCE study protocol was approved by the Institutional Review Board at each site and was performed in accordance with the International Conference on Harmonization Guidelines for Good Clinical Practice and the Declaration of Helsinki. All patients provided written informed consent before entering the study.

### Outcome measures

The primary endpoint was ARR at Week 48, as reported previously [3]. Relapses were defined as new or recurrent neurologic symptoms not associated with fever or infection, lasting ≥24 h, and accompanied by new objective neurologic findings upon evaluation by the examining neurologist. Relapses were confirmed by an Independent Neurology Evaluation Committee (INEC). Standardized neurological assessments including EDSS were performed at baseline, every 12 weeks, and at the time of suspected relapse. MRI scans were performed at screening and at Weeks 24, 48, and 96, and were assessed centrally by an expert reader blinded to treatment allocation.

### Statistical analyses

In the primary analysis, ARR (total number of relapses divided by patient-years in the study, excluding data obtained after patients switched to alternative multiple sclerosis drugs) was analyzed for the intent-to-treat (ITT) population with a negative binomial regression model adjusted for baseline EDSS score (<4 vs ≥4), baseline relapse rate (number of relapses in 3 years before study entry divided by 3), and age (<40 vs ≥40 years). Subgroup and sensitivity analyses were performed as follows.

#### Subgroup analyses

ARR was evaluated in pre-specified analyses at Year 1, and repeated at 2 years, among subgroups based on baseline demographics and disease characteristics. The baseline demographics subgroups assessed included: gender, age (<40 and ≥40 years), weight (by quartiles), and geographical regions [Region 1 (Western Europe and North America): Belgium, France, Germany, The Netherlands, Spain, United Kingdom, the United States, and Canada; Region 2 (Eastern Europe): Bulgaria, Croatia, Czech Republic, Estonia, Greece, Latvia, Poland, Romania, Russian Federation, Serbia, and Ukraine; Region 3 (South and Central America, Asia, and Australia): Chile, Colombia, Mexico, Peru, Georgia, India, and New Zealand]. Regions were grouped based on similarities in healthcare delivery system. Disease characteristic subgroups examined included: relapses during the previous 3 years (2, >2); McDonald criteria (1 vs 2, 3, 4); prior MS treatment (“Yes” vs “No”); EDSS score (<4 vs ≥4); T2 hyperintense lesion volume (above or below median); and presence of gadolinium-enhancing (Gd+) lesions (present vs absent).

Year 1 subgroup analyses of ARR were performed in ITT population, for peginterferon beta-1a every 2 weeks or every 4 weeks compared with placebo. Beyond Year 1, no patients remained on placebo. Subgroup analyses of ARR over 2 years were performed in the ITT population, and results are presented for patients who had received continuous active treatment from baseline, with comparison between peginterferon beta-1a every-2-weeks dosing and every-4-weeks dosing.

#### Sensitivity analyses

Five sensitivity analyses were conducted for the primary endpoint to compare ARR in peginterferon beta-1a every 2 weeks and every 4 weeks versus placebo groups at Year 1. The analyses of ARR included: (1) the per-protocol population; (2) the ITT population using a Poisson regression model (whereas the primary analysis used a negative binomial regression model); (3) the ITT population including all relapses reported on the unscheduled relapse assessment visit (whereas the primary analysis included INEC-confirmed relapses only); (4) the ITT population based on protocol-defined objective relapses; and (5) the ITT population using a negative binomial regression model with presence/absence of baseline Gd+ lesions as a covariate in the model (as well as baseline EDSS, baseline relapse rate, and age, which were the factors included in the model in the primary analysis). Similar sensitivity analyses were performed over 2 years for patients who had received continuous active treatment from baseline, with comparison between every-2-weeks dosing and every-4-weeks dosing.

## Results

### Baseline disposition and characteristics

A total of 1512 patients were randomized and received treatment with placebo (*n* = 500), peginterferon beta-1a 125 mcg every 2 weeks (*n* = 512) or peginterferon beta-1a 125 mcg every 4 weeks (*n* = 500). The ITT population included all patients who were randomized and received at least 1 active dose of study treatment. This population was used for all analyses in this manuscript, using available data until the time of dropout for patients dropping out prior to the end of Year 2.

As reported previously [3, 4], baseline demographics and disease characteristics were well balanced across the treatment groups (Table 1). Year 1 of the study was completed by 86, 88, and 91 % of patients receiving peginterferon beta-1a every 2 weeks, peginterferon beta-1a every 4 weeks, and placebo, respectively. Year 2 of the study was completed by 89, 94, 88, and 86 % of patients in continuous treatment every 4 weeks and every 2 weeks and delayed treatment every 4 weeks and every 2 weeks, respectively. Baseline characteristics did not differ significantly across regions, and clinical behavior was similar across different regions (data not shown).

**Table 1:** Baseline demographic, disease, and MRI characteristics

	Placebo (*n* = 500)	Peginterferon beta-1a 125 mcg every 2 weeks *(n* = 512)	Peginterferon beta-1a 125 mcg every 4 weeks (*n* = 500)
Age (years)	36.3 (9.7)	36.9 (9.8)	36.4 (9.9)
Women, *n* (%)	358 (72)	361 (71)	352 (70)
Weight (kg)	69.2 (16.2)	69.6 (17.4)	68.3 (14.6)
White ethnic origin, *n* (%)	412 (82)	416 (81)	409 (82)
Geographic region, *n* (%)			
India	56 (11)	58 (11)	56 (11)
North America	17 (3)	19 (4)	16 (3)
Western Europe	38 (8)	41 (8)	39 (8)
Eastern Europe	354 (71)	355 (69)	355 (71)
Rest of the world	35 (7)	39 (8)	34 (7)
Time since first MS symptoms (years)	6.3 (6.3)	6.9 (6.6)	6.5 (6.1)
Time since MS diagnosis (years)	3.5 (4.6)	4.0 (5.1)	3.4 (4.4)
Relapses within the previous 3 years	2.6 (1.00)	2.6 (0.99)	2.5 (0.77)
Relapses within the previous 12 months	1.6 (0.67)	1.6 (0.67)	1.5 (0.62)
EDSS score	2.44 (1.18)	2.47 (1.26)	2.48 (1.24)
<4, *n* (%)	432 (86)	423 (83)	413 (83)
≥4, *n* (%)	68 (14)	89 (17)	87 (17)
Patients without Gd+ lesions, *n* (%)	296 (59)	334 (65)	297 (59)
Number of Gd+ lesions	1.6 (3.8)	1.2 (3.4)	1.8 (5.4)
Number of T2 lesions	50.6 (35.7)	48.7 (36.8)	51.4 (36.0)
Volume of T2 lesions, cm^3^	10.1 (11.9)	9.8 (11.6)	11.3 (13.2)
Previous treatment^a^, *n* (%)			
Glatiramer acetate	24 (5)	27 (5)	28 (6)
Interferon beta-1b	6 (1)	8 (2)	5 (1)
Interferon beta-1a	5 (1)	4 (<1)	6 (1)
Other	58 (12)	56 (11)	58 (11)

Data are mean (SD) or *n* (%)

*EDSS* Expanded Disability Status Scale*, Gd*+ gadolinium-enhancing*, MRI* magnetic resonance imaging, *MS* multiple sclerosis

^a^Patients who had taken more than one drug were counted more than once: the total number of patients who had previously been treated was 260 (17 %)

### Primary efficacy results

**Fig. 1:**
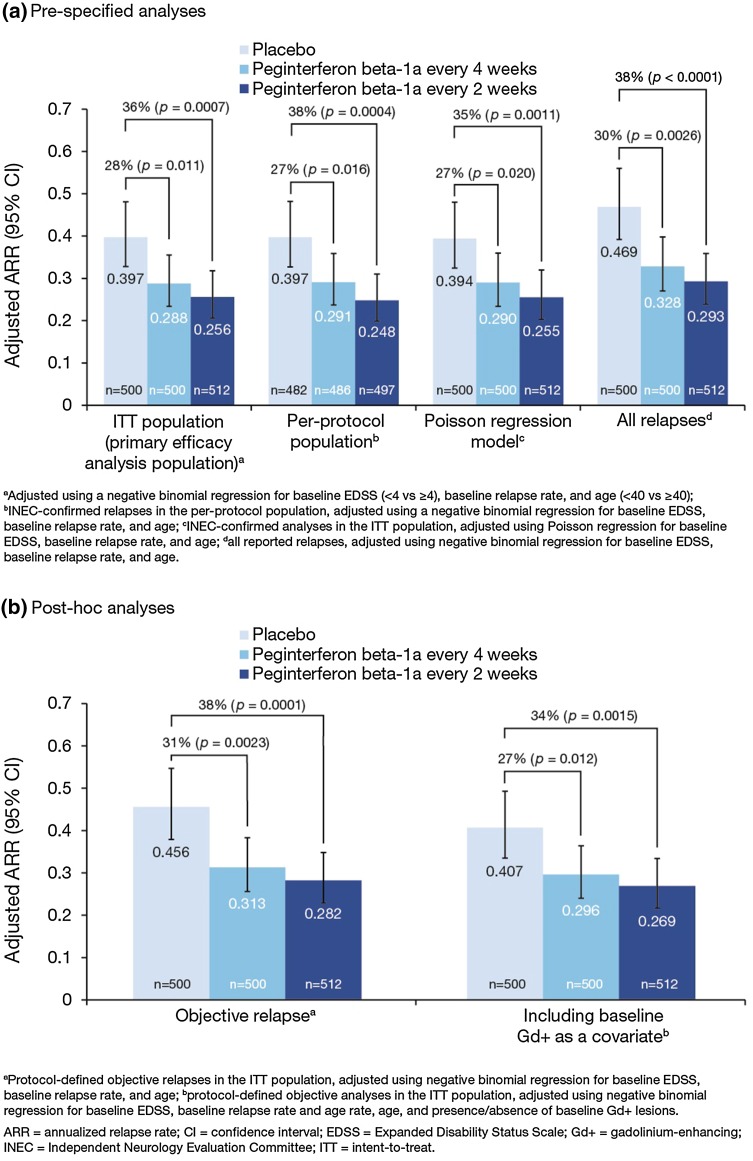
Pre-specified **a** and post hoc **b** sensitivity analyses for ARR at Year 1

The ARR at Year 1 was 0.397 relapses per patient-year (95 % CI 0.328–0.481) in the placebo group, 0.256 (0.206–0.318) in the every 2 weeks group, (36 % lower, *p* = 0.0007) and 0.288 (0.234–0.355) in the every 4 weeks group (28 % lower, *p* = 0.011, Fig. 1) [3]. Compared with Year 1, ARR was further reduced in Year 2 with continued every-2-week dosing (from 0.256 to 0.178) and maintained with continued every-4-week dosing (0.288 vs 0.291) [4].

### Subgroup analyses of ARR by baseline demographic characteristics

**Fig. 2:**
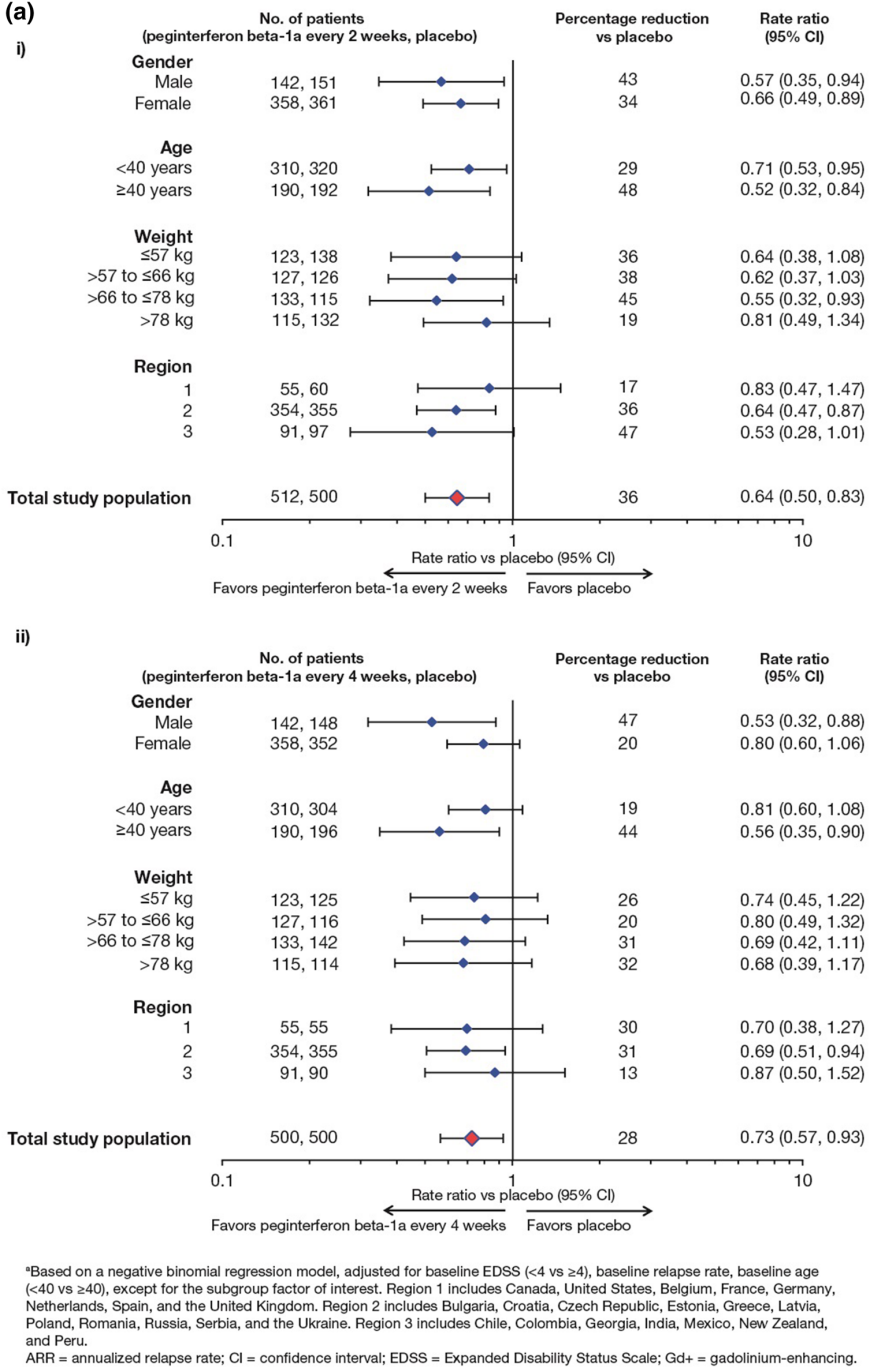

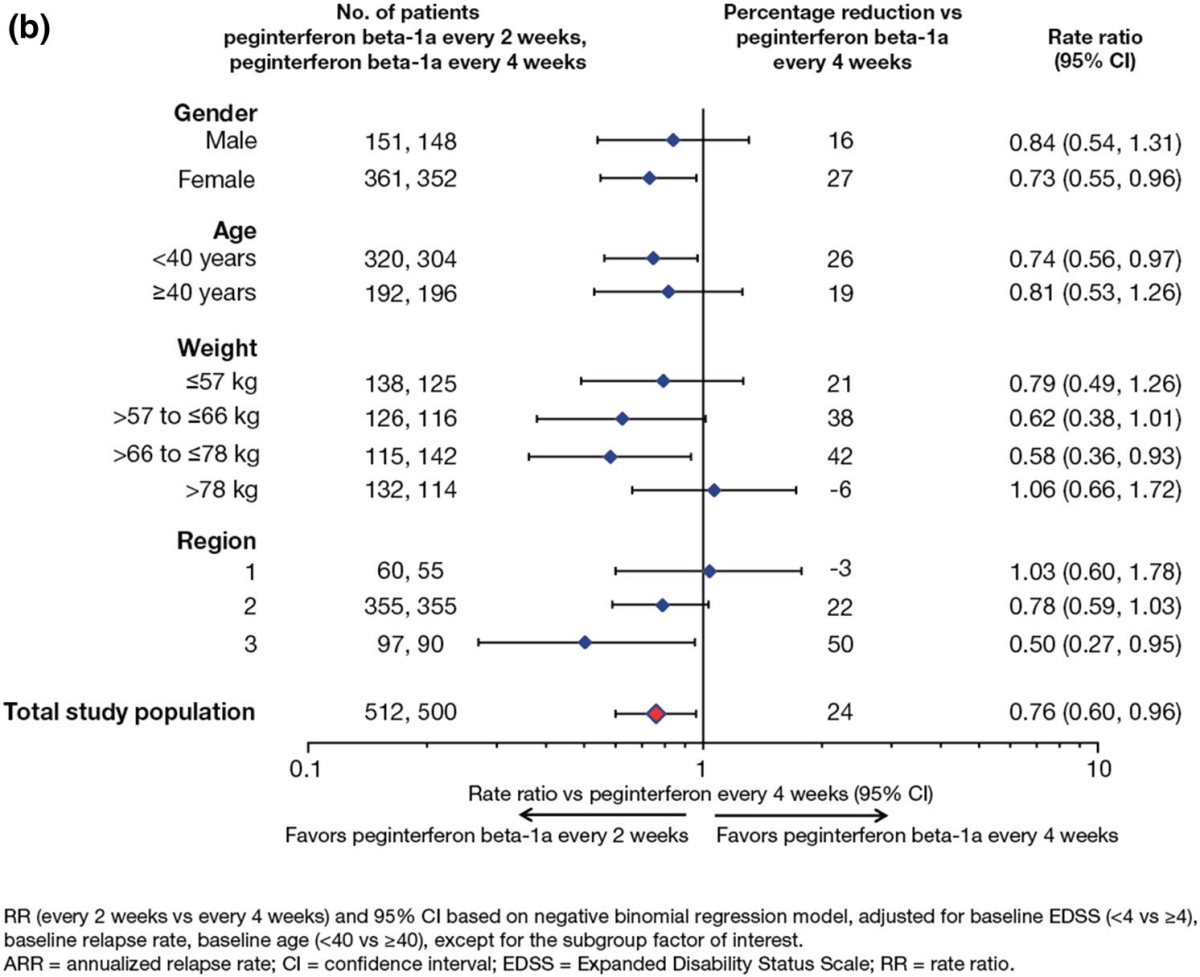
Analyses^a^ of adjusted annualized rate of relapse by baseline demographic characteristics: **a** at Year 1 (peginterferon beta-1a every 2 weeks vs placebo *i* and peginterferon beta-1a every 4 weeks vs placebo *ii*); **b** over 2 years (peginterferon beta-1a every 2 weeks vs every 4 weeks)

ARR at Year 1 and over 2 years by baseline demographic characteristics is shown in Fig. 2. At Year 1, significant reduction in ARR was evident with peginterferon beta-1a every 2 weeks compared with placebo, regardless of age and gender. All of the subgroups stratified by weight or grouped by geographical region showed numerical reductions in ARR with peginterferon beta-1a every 2 weeks versus placebo, with some subgroups reaching significance (Fig. 2a, i). Effects of peginteferon beta-1a every 4 weeks showed similar trends to peginterferon beta-1a every 2 weeks across subgroups defined by demographic characteristics at Year 1 (Fig. 2a, ii). In the over-2-years analyses, the ARR achieved with peginterferon beta-1a every 2 weeks was numerically lower than with peginterferon beta-1a every 4 weeks for most of the subgroups analyzed (Fig. 2b).

### Subgroup analyses of ARR by baseline disease characteristics

**Fig. 3:**
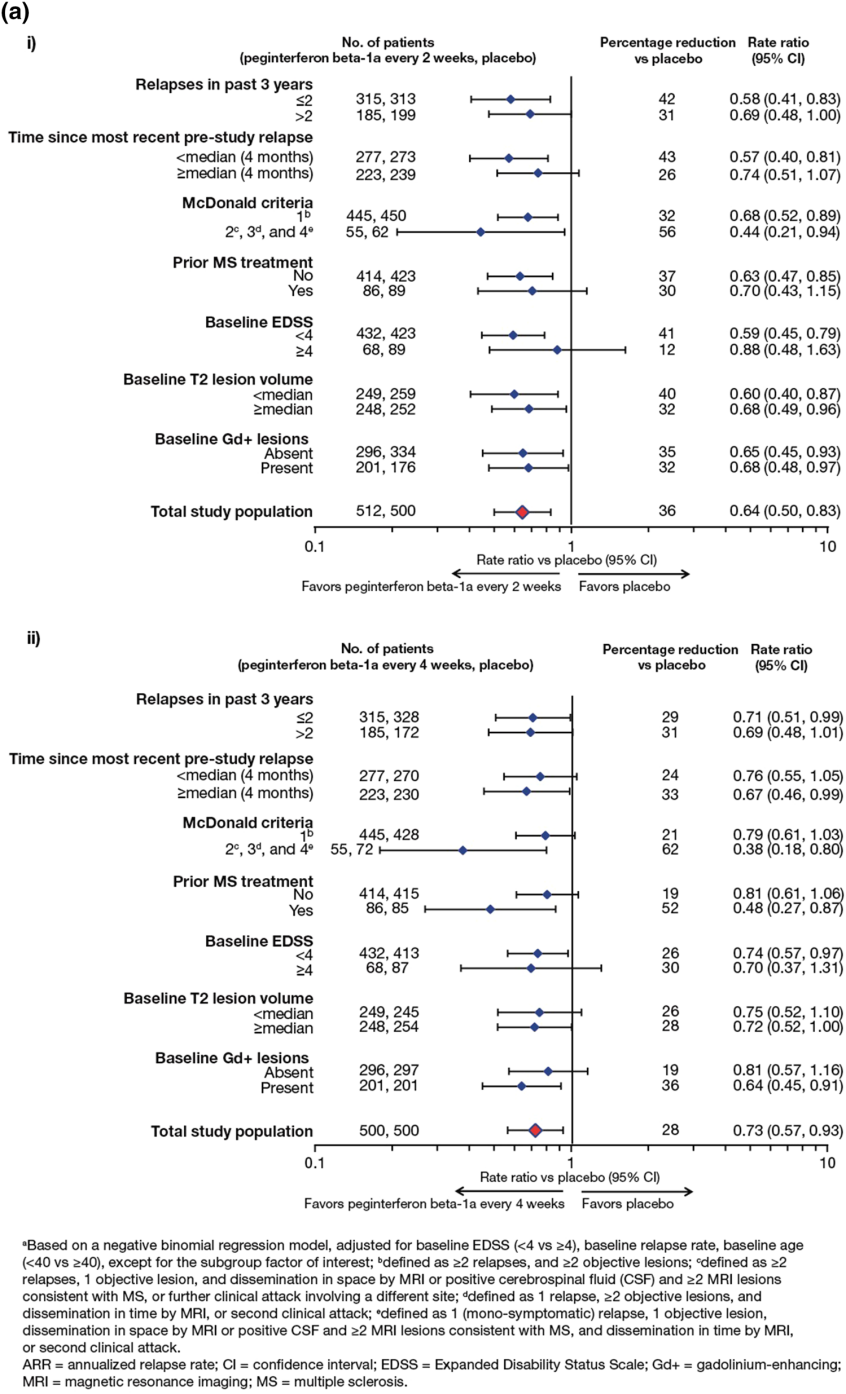

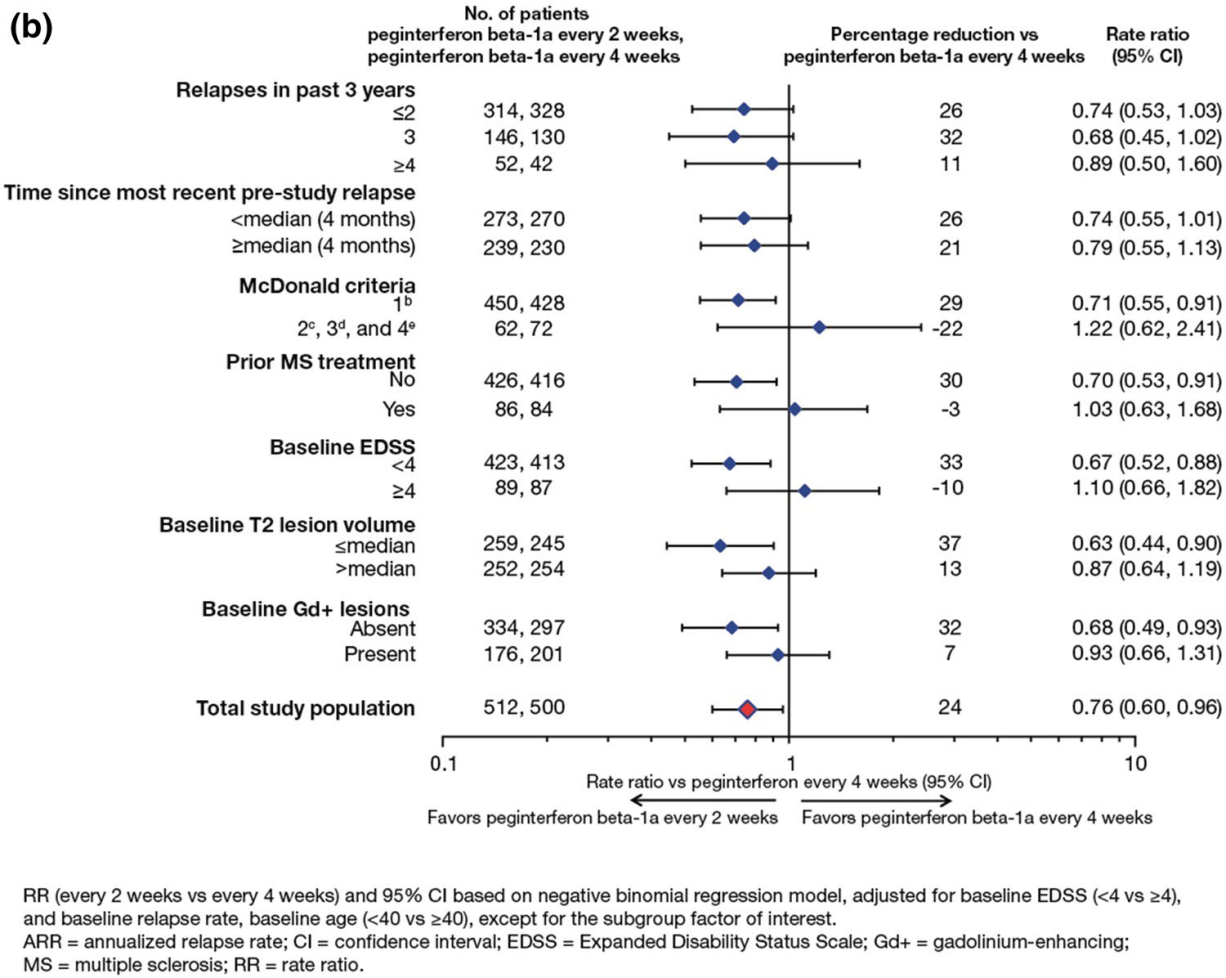
Analyses^a^ of adjusted annualized rate of relapse by baseline disease characteristics: **a** at Year 1 (peginterferon beta-1a every 2 weeks vs placebo *i* and peginterferon beta-1a every 4 weeks vs placebo *ii*); **b** over 2 years (peginterferon beta-1a every 2 weeks vs every 4 weeks)

ARR at Year 1 and over 2 years by baseline disease characteristic subgroups is shown in Fig. 3. At Year 1, peginterferon beta-1a every 2 weeks produced significant reductions in ARR compared with placebo across most of the subgroups evaluated according to baseline disease characteristics (Fig. 3a, i). Peginterferon beta-1a every 4 weeks showed similar trends, with consistent numerical reductions in ARR compared with placebo, although significance was reached in fewer subgroups than with peginterferon beta-1a every 2 weeks (Fig. 3a, ii). ARR over 2 years was numerically lower in patients receiving peginterferon beta-1a every 2 weeks versus every 4 weeks across most baseline disease characteristics subgroups evaluated, reaching significance in some of the largest subgroups in which variation was smaller, such as treatment-naïve patients, those meeting McDonald criterion 1, and those with baseline EDSS score < 4 (Fig. 3b).

### Sensitivity analyses in the overall study population

**Fig. 4:**
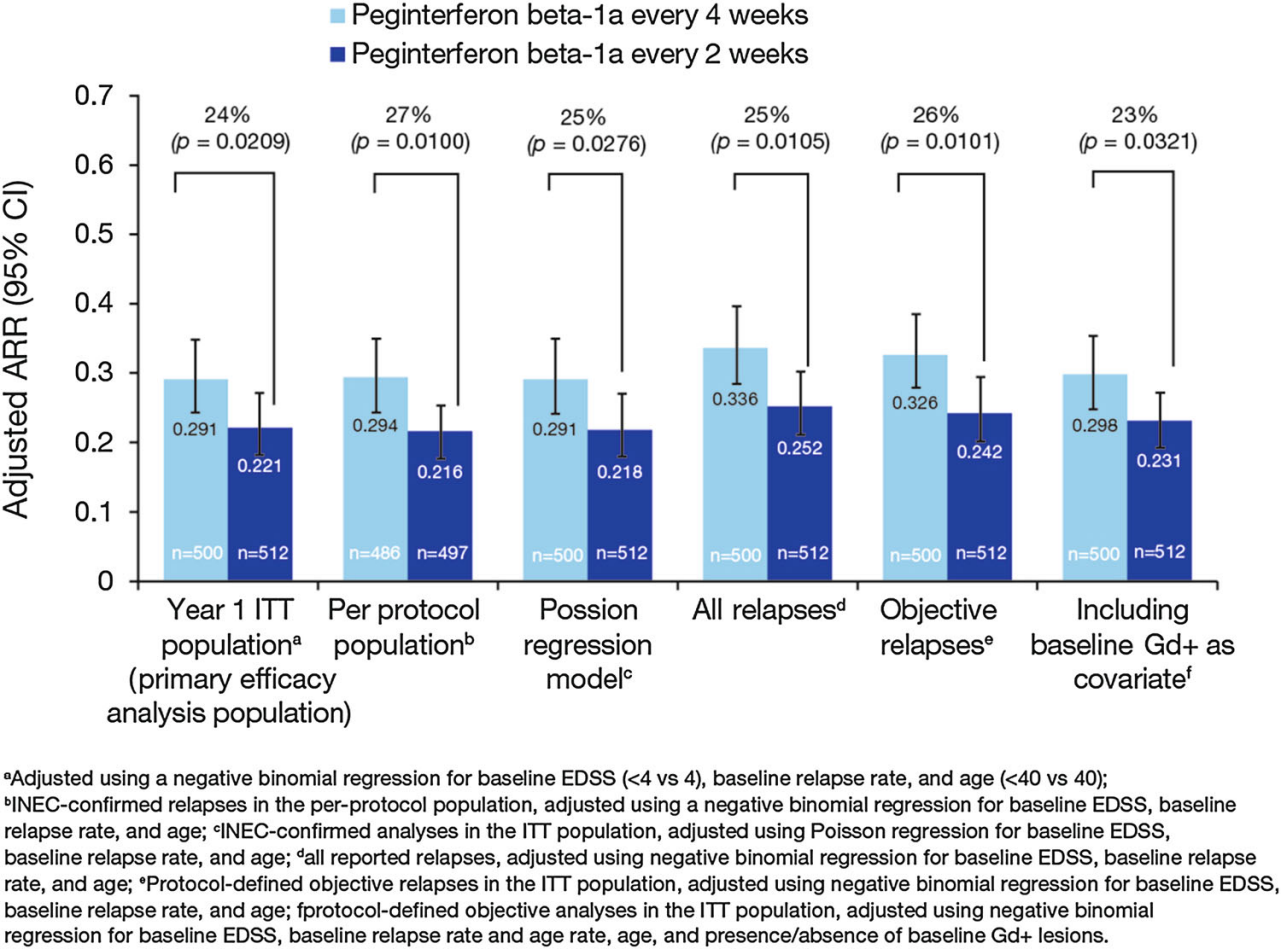
Post-hoc sensitivity analyses for ARR over 2 years

Results from the sensitivity analyses at Year 1 were consistent with the primary analysis as reported by Calabresi et al. [3] (Fig. 1). Compared with placebo, ARR was significantly reduced in the peginterferon beta-1a every-2-weeks (34–38 %) and every-4-weeks (27–31 %) groups. Sensitivity analyses over 2 years are shown in Fig. 4. Compared with peginterferon every 4 weeks, ARR was significantly reduced (22–27 %) in the peginterferon beta-1a every-2-weeks group.

## Discussion

In the ADVANCE trial, subgroup analysis of the primary endpoint, ARR, across clinically relevant subpopulations of RRMS patients with varied baseline demographics and disease characteristics, found that peginterferon beta-1a treatment demonstrated generally consistent benefits in terms of ARR, with results that were similar to those observed in the primary analysis. These findings suggest that, among the general RRMS population, most subgroups of patients could derive benefit from treatment with peginterferon beta-1a.

There was considerable overlap in the 95 % confidence intervals (CI) for rate ratios for peginterferon beta-1a versus placebo in Year 1 across the examined subgroups (although some point estimate differences across subgroups were observed), indicating that peginterferon beta-1a had similar efficacy for reduction in ARR regardless of gender, age, weight, geographical region, and baseline disease characteristics. While some subgroups failed to reach statistical significance for peginterferon beta-1a versus placebo (as indicated by 95 % CI for rate ratios crossing 1), this tended to be in smaller subgroups which exhibited a higher degree of variability, and so may be related to reduced statistical power in small subgroups. For example, stratification by weight produced 4 smaller subgroups, which had wider 95 % CIs than larger subgroups in the binary categories such as age and gender; among the geographical regions, Region 2 (Eastern Europe) was the largest subgroup and had the tightest 95 % CIs, revealing significant effects of both peginterferon beta-1a every 2 weeks and every 4 weeks compared with placebo. Similarly, over 2 years, there were some differences in ARR among the subgroups, but the overlap in 95 % CIs suggested that the treatment effect of peginterferon beta-1a every 2 weeks on ARR was greater than with every-4-weeks dosing in most subgroups defined by baseline demographic characteristics. Among subgroups defined by baseline disease characteristics, peginterferon beta-1a treatment every 2 weeks over 2 years demonstrated generally consistent efficacy versus every 4 weeks on ARR (the primary endpoint).

Sensitivity analyses, which examine the influence of protocol design errors, unintended biases, and deviations from assumptions of underlying models, are designed to assess whether a primary endpoint result is robust and generalizable. The current sensitivity analyses demonstrated that the primary endpoint of the ADVANCE study was robust across 2 years, with little difference in outcomes across several pre-planned and post hoc analyses.

## Conclusion

Peginterferon beta-1a demonstrated generally consistent benefits on the primary endpoint of ADVANCE, ARR at Year 1, across clinically relevant subgroups of RRMS patients with varied baseline demographic and disease characteristics. Similar results were observed over 2 years. Overall, these subgroup and sensitivity analyses support the results from the primary analysis, indicating that peginterferon beta-1a has the potential to provide benefits to patients with MS, with a convenient, low-frequency, SC dosing regimen.
